# Optimizing antiresorptive treatment in patients with bone metastases: time to initiation, switching strategies, and treatment duration

**DOI:** 10.1007/s00520-019-04676-6

**Published:** 2019-02-13

**Authors:** AnneMarthe Mjelstad, Gustav Zakariasson, Antonis Valachis

**Affiliations:** 1 Faculty of Medicine, Uppsala University, Uppsala, Sweden; 2 Department of Oncology, Faculty of Medicine and Health, Örebro University, SE 70182 Örebro, Sweden; 3 Department of Oncology Sörmland, Mälarsjukhuset, Eskilstuna, Sweden

**Keywords:** Bisphosphonate, Denosumab, Treatment initiation, Duration, Switching

## Abstract

**Purpose:**

The aim of this study was to investigate the optimal use of antiresorptive therapy in patients with metastatic cancer in terms of time to treatment initiation, switching strategy in case of skeletal-related event (SRE) or skeletal disease progression, and treatment efficacy beyond 2 years.

**Methods:**

We conducted a single-center retrospective cohort study including consecutive cancer patients with bone metastases that have received antiresorptive treatment between 2009 and 2015. The outcomes of interest were the time to first and subsequent symptomatic skeletal event (SSE), the skeletal morbidity rate, and the incidence of antiresorptive therapy-specific adverse events depending on the research question.

**Results:**

In total, 255 patients included in our study cohort. The time to treatment initiation (direct (*n* = 143 patients) vs. delayed (*n* = 87 patients) defined as > 3 months after diagnosis of bone metastases) was not found to influence the time to SSE in (hazard ratio (HR) 0.93; 95% confidence interval (CI) 0.65–1.34) with comparable toxicity. Switching strategy after first SRE or due to skeletal disease progression from bisphosphonates to denosumab was independently associated with longer time to SRE (HR 0.47, 95% CI 0.25–0.88, *p* value = 0.019) compared with continuation with the same bisphosphonate. Using the landmark approach at 24 months and including 121 patients that survived for more than 2 years, we found that treatment continuation beyond 2 years was associated with longer time to first SSE after 2 years (HR 0.41; 95% CI 0.19–0.93).

**Conclusions:**

Our hypothesis-generating results support a more individualized approach on antiresorptive treatment including the lack of detrimental effect when the treatment is delayed, the potential benefit of switching strategy after skeletal disease progression or SSE, and the benefit of continuing antiresorptive treatment beyond 2 years.

## Introduction

Bone is a common site of metastasis that occur in most tumor types, especially in breast, prostate, and lung cancer [1, 2]. The presence of bone metastases can lead to complications known as skeletal-related events (SRE) such as pathological fractures, spinal cord compression, need of radiation therapy due to bone pain, hypercalcemia of malignancy, and need of orthopedic or neurosurgical procedures [1]. These events can negatively affect patients’ quality of life [3] and have been associated with increased mortality [4–6] and increased resource utilization [7].

Treatment with antiresorptive agents, including bisphosphonates or denosumab, is highly recommended in cancer patients with bone metastases, since it reduces the risk of SRE [8, 9]. However, several issues on the optimal use of antiresorptive agents in respect of time to initiation, therapeutic strategy after a SRE while on antiresorptive treatment, and duration of treatment remain unclear due to the paucity of evidence. The current international guidelines recommend that therapy with an antiresorptive agent should be initiated in the presence of a documented metastatic bone lesion, a switching strategy from bisphosphonates to denosumab can be considered if a SRE occurs while on bisphosphonate therapy, whereas the treatment duration should be life-long until impairment in the patient’s general performance status or occurrence of severe adverse events [10–13]. The expert panels recognize, however, that these recommendations are based on scarce evidence [10–13].

The purpose of the present study was to investigate the efficacy of antiresorptive treatment in patients with direct initiation of the therapy compared with patients with delayed initiation, the efficacy of switching antiresorptive agents due to symptomatic skeletal event (SSE) or skeletal disease progression, and whether cancer patients with bone metastases benefit from treatment with antiresorptive agents for a period of more than 2 years.

## Patients and methods

### Study population

We performed a single-center retrospective cohort study. We identified all cancer patients with bone metastases that have received antiresorptive treatment (bisphosphonates and/or denosumab) as metastatic treatment strategy between 2009 and 2015 at the Department of Oncology in Eskilstuna, Sweden.

The eligible patients were identified through searching the electronic database for oncologic treatment that is used at the Department of Oncology in Eskilstuna (MOSAIQ Oncology Information System).

We included all patients that fulfilled the following inclusion criteria: (1) cancer patients with bone metastases that have received antiresorptive treatment as metastatic treatment strategy; (2) antiresorptive therapy could include any of the following regimens: zoledronic acid, pamidronate, ibandronate, or the non-bisphosphonate antiresorptive regimen denosumab; (3) at least 3 consecutive months of antiresorptive therapy were required.

We excluded patients with hematological malignancies; patients who received antiresorptive treatment as adjuvant therapy; patients who received antiresorptive agents other than zoledronic acid, pamidronate, ibandronate, or the non-bisphosphonate antiresorptive regimen denosumab; and patients who received antiresorptive treatment for < 3 months.

### Data collection

The following data were extracted from electronic medical records: age at diagnosis, comorbidities (with special interest on presence of osteoporosis, other risk factors for osteoporosis, prior bisphosphonate therapy for non-cancer indications), date at diagnosis; cancer type; date at diagnosis of bone metastasis, number of bone metastases, type of bone metastases (sclerotic, lytic, mixed), metastases to other sites; date at initiation of antiresorptive treatment, type of antiresorptive treatment, number of cycles with antiresorptive treatment; switching of antiresorptive agent (date and reason), date and type of SRE, date and type of adverse event (osteonecrosis of the jaw (ONJ), hypocalcaemia, renal impairment); death, time to death, and cause of death.

### Outcomes and definitions

For the research question on the time to treatment initiation, the patients were categorized as direct initiation of antiresorptive treatment if the start of treatment was initiated within 3 months from the radiological diagnosis of bone metastases and as delayed initiation if the start of treatment was delayed for at least 3 months from bone metastasis diagnosis.

The primary outcome was the time to first SSE with different definitions of time zero for each research question. For the analysis of the time to initiation, we used as time zero the time from diagnosis of bone metastasis. For the analysis of switching strategies, we used as time zero the time from first SRE recorded or the time for the first disease progression to the bones. For the analysis of the prolonged duration, we used as time zero the landmark time, namely 2 years (a detailed description on the statistical analysis subsection).

A SSE was defined as including one or more of the following: pathological fracture, spinal cord compression, need of radiation therapy due to bone pain, hypercalcemia of malignancy, and need of orthopedic or neurosurgical procedures. Hypercalcemia of malignancy will be considered SRE in case of hospitalization (grade 3: corrected calcium > 3.1–3.4 or ionized calcium > 1.5–1.6; grade 4: corrected calcium > 3.4 or ionized calcium > 1.6). A 21-day window was used to identify a new event after a previous SSE. We defined our skeletal events as SSE rather than SRE because we did not prospectively collect all the SREs. The SSE events were captured either during the laboratory and radiological evaluation of cancer treatment or due to symptoms that led patients presenting to the hospital for evaluation.

Secondary outcomes included the time to subsequent SSE defined as the time from the first SSE to the subsequent SRE occurring > 21 days after the previous one, the skeletal morbidity rate (SMR, defined as the ratio of the number of SSEs for each subject divided by the subject’s time at risk in years), and the incidence of antiresorptive therapy-specific adverse events (ONJ, hypocalcaemia, renal impairment) during treatment duration.

All antiresorptive therapy-specific adverse events were classified according to the National Cancer Institute Common Terminology Criteria for Adverse Events version 4.0 [14]. In particular, renal impairment was classified as grade 1 if creatinine was increased 1.5–2.0 × above baseline, grade 2 if creatinine was increased 2–3 × above baseline, grade 3 if creatinine was increased > 3 × baseline and led to hospitalization, and grade 4 in life-threatening consequences where dialysis was indicated. Hypocalcemia was classified according to the ionized calcium level (grade 1: ionized calcium lower limit normal – 1.0 mmol/L; grade 2: ionized calcium < 1.0–0.9 mmol/L with symptoms; grade 3: ionized calcium < 0.9–0.8 mmol/L when hospitalization was indicated; grade 4: ionized calcium < 0.8 mmol/L with life-threatening consequences). ONJ was classified as grade 1 in asymptomatic cases when intervention is not indicated, grade 2 in symptomatic cases when medical intervention was indicated, grade 3 in cases with severe symptoms, with limiting self-care ADL when elective operative intervention was indicated, and grade 4 in cases with life-threatening consequences when urgent intervention was indicated. Oral supplementation with vitamin D and calcium was prescribed to all the patients treated with antiresorptive therapy.

The study has been approved by the Regional Ethical Review Board in Stockholm region (Dnr: 2017/1640–31).

### Statistical analysis

Categorical variables were summarized by the number and percentage of patients in each category whereas continuous variables were summarized by mean, median, minimum, and maximum values.

For time-to-event outcome (time to first SSE, time to subsequent SSE) the potential association between variables and outcome of interest was assessed by the Kaplan-Meier method (logrank test for statistical significance). The variables with < 0.1 level of significance in the bivariate analysis were included in the Cox proportional hazards models. Three different models were constructed for the three different research questions. To avoid immortal time bias for the analysis of prolonged antiresorptive treatment, we used the landmark approach using 2 years as landmark time [15].

Confidence intervals (95% CI) for the percentage of patients experiencing adverse events were calculated using the Wilson score method. The incidences of adverse events were compared between the different subgroups (direct vs. delayed antiresorptive treatment; prolonged vs. no prolonged treatment) using chi-square or Fisher’s exact test.

All reported *p* values of statistical tests were two-tailed, and *p* < 0.05 was taken to be statistically significant. All analyses were performed using the SPSS.

## Results

### Study cohort

**Fig. 1:**
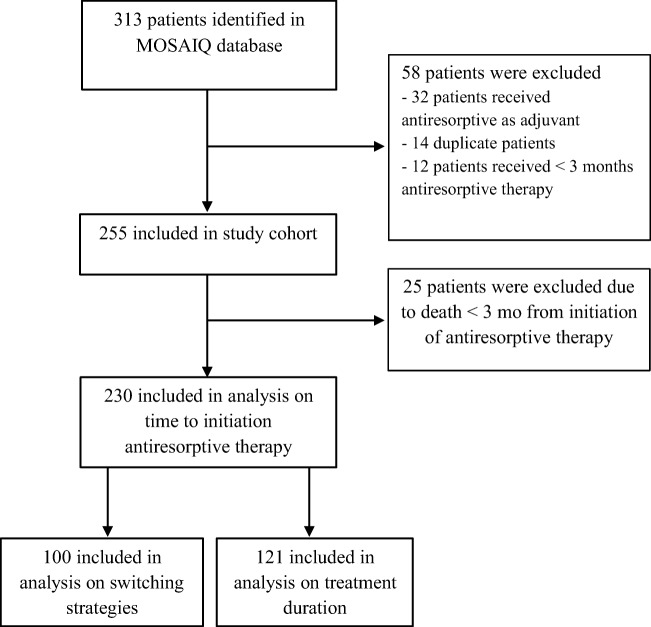
Flowchart diagram of patient selection

In total, 313 cancer patients treated with antiresorptive therapy were identified through searching of electronic database. Fifty-eight patients were excluded according to our exclusion criteria (Fig. 1). The study cohort included 255 patients, with a median age of 67 years (range: 25–98) and a median Charlson comorbidity index of 3 (range 0–8) (Table 1). The most common type of cancer was breast cancer (37.3%) followed by prostate cancer (32.9%).

**Table 1:** Characteristics of study cohort

	*N* (%)
Sex	
Female	132 (51.8)
Male	123 (48.2)
Age at diagnosis, median (range)	67 (25–98
Charlson score at diagnosis, median (range)	3 (0–8)
Smoking	
Non-smoker	85 (33.3)
Former smoker	49 (19.2)
Current smoker	33 (12.9)
Data missing	88 (34.5)
Alcohol consumption	
None	5 (2.0)
Low	5 (2.0)
Moderate	11 (4.3)
High	2 (8.0)
Data missing	232 (91.0)
BMI at diagnosis	
< 20	12 (4.7)
20–25	74 (29.0)
25–30	81 (31.8)
> 30	47 (18.4)
Data missing	41 (16.1)
Primary cancer	
Breast cancer	95 (37.3)
Prostate cancer	84 (32.9)
Lung cancer	29 (11.4)
Urogenital cancer (not prostate)	16 (6.3)
Other	31 (12.2)
PS at treatment start	
0	99 (38.8)
1	59 (23.1)
2	43 (16.9)
3	24 (9.4)
4	4 (1.6)
Data missing	26 (10.2)
Location of metastases	
Bone only	98 (38.4)
Bone + other location	157 (61.6)
Type of bone metastases	
Sclerotic	132 (51.8)
Lytic	57 (22.4)
Mixed	66 (25.9)
Location of bone metastases	
Vertebrae and/or thorax	236 (92.5)
Pelvis	161 (63.1)
Lower extremity	51 (20.0)
Upper extremity	19 (7.5)
Scull	18 (7.1)

Bone-only metastases were identified in 98 patients (38.4%). The distribution of bone metastatic type among lytic, sclerotic, and mixed was 51.8%, 22.4%, and 25.9%, respectively. The most common skeletal metastatic site was vertebral or thorax (92.5%), followed by metastases in the pelvis (63.1%).

The type of antiresorptive treatment used and type of SSE are presented in Table 2. Zoledronic acid was the most common type of antiresorptive therapy (58.8%) as first line whereas denosumab was the most common treatment as second line (72.0%). The most common reason for antiresorptive treatment discontinuation was patient death (31.8%).

**Table 2:** Type of antiresorptive treatment, reason for treatment discontinuation, and type of skeletal-related events in study cohort

	*N* (%)
Antiresorptive treatment	
1st treatment	
Zoledronic acid	150 (58.8)
Ibandronate (iv)	96 (37.6)
Ibandronate (po)	5 (2.0)
Denosumab	4 (1.6)
Reason for discontinuation of 1st treatment	
Patient died	81 (31.8)
Disease progression	49 (19.2)
Physician’s choice	37 (14.5)
Toxicity	31 (12.2)
Planned	18 (7.1)
Impaired performance status	25 (9.1)
Patient’s choice	4 (1.6)
Unknown	10 (3.9)
2nd treatment	
Zoledronic acid	15 (22.4)
Ibandronate (iv)	9 (13.4)
Ibandronate (po)	1 (1.5)
Denosumab	42 (50.0)
Reason for discontinuation of 2nd treatment	
Patient died	2 (3.0)
Disease progression	6 (9.0)
Physician’s choice	15 (22.4)
Toxicity	6 (9.0)
Planned	2 (3.0)
Impaired performance status	8 (11.9)
Patient’s choice	3 (4.5)
Unknown	0 (0.0)
Type of SRE	
1st SRE	
Radiotherapy due to pain	84 (32.9)
Pathological fracture	28 (20.6)
Radiotherapy due to immediate risk for fracture	10 (7.4)
Spinal cord compression	10 (7.4)
Surgery to bone due to fracture	2 (3.1)
Hypercalcemia of malignancy	3 (2.3)
Total number of SREs	136 (53.3)
2nd SRE	
Radiotherapy due to pain	38 (58.5)
Pathological fracture	13 (20.3)
Spinal cord compression	7 (10.9)
Radiotherapy due to immediate risk for fracture	4 (6.2)
Surgery to bone due to fracture	2 (3.1)
Hypercalcemia of malignancy	0 (0.0)
Total number of SREs	65 (25.1)
3rd SRE	
Radiotherapy due to pain	21 (75.0)
Pathological fracture	4 (14.3)
Spinal cord compression	1 (3.6)
Radiotherapy due to immediate risk for fracture	1 (3.6)
Surgery to bone due to fracture	0 (0.0)
Hypercalcemia of malignancy	1 (3.6)
Total number of SREs	28 (11.0)
4th SRE	
Radiotherapy due to pain	9 (90.0)
Pathological fracture	0 (0.0)
Spinal cord compression	1 (10.0)
Radiotherapy due to immediate risk for fracture	0 (0)
Surgery to bone due to fracture	0 (0.0)
Hypercalcemia of malignancy	0 (0.0)
Total number of SREs	10 (3.9)

Radiotherapy due to pain was the most common type of first and subsequent SSE followed by pathological fracture. A detailed description of different types of SSEs is presented in Table 2.

### Time to treatment initiation

The research question on time to treatment initiation was studied in 230 patients who survived more than 3 months from diagnosis of bone metastases. The cohort was divided into two groups depending on time to antiresorptive treatment initiation in direct treatment group (*n* = 143 patients; 62.2%) and delayed treatment group (*n* = 87 patients; 37.8%). The median time to treatment initiation for the direct group was 1 month (range 0–3 months) whereas for the delayed treatment group was 7 months (range 3–15 months).

**Fig. 2:**
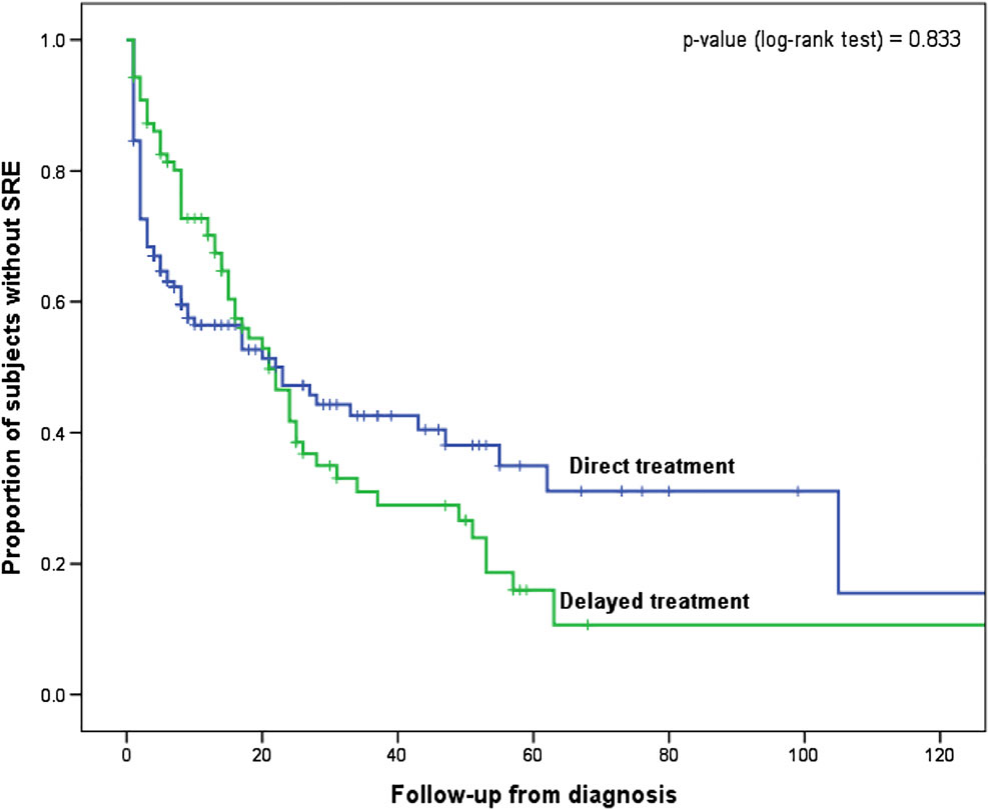
Kaplan-Meier curve for time from diagnosis of bone metastasis to first skeletal-related event (SRE) in direct antiresorptive treatment group vs. delayed treatment group

No statistically significant difference was observed between the direct and the delayed group in terms of time to SSE (Fig. 2.) with *p* value = 0.833. A Cox proportional hazards model was also performed including time to treatment initiation as depending variables along with other variables with potential clinical importance on the risk for SRE (age, SSE at diagnosis, number and type of bone metastasis). The time to treatment initiation was not found to influence the time to SRE in multivariate analysis either (HR 0.93; 95% CI 0.65–1.34). No difference in SMR between the two treatment groups was observed (0.75 (standard deviation, SD 1.08) SSE/year for direct group vs. 0.72 (SD 0.86) SSE/year for delayed group, *p* value = 0.827).

The toxicity rate for antiresorptive treatment-specific toxicity was comparable between the direct and delayed groups with 43% vs. 34.5% renal toxicity (*p* value = 0.214), 41.5% vs. 48.8% hypocalcaemia (*p* value = 0.268), and 4.2% vs. 5.7% ONJ (*p* value = 0.752). In total, 11 patients (4.8%) developed ONJ. Most of the patients developed grade 1–2 renal toxicity with 5 patients (3.5%) with grade 3 renal toxicity in the direct group and 3 patients (3.4%) in the delayed group whereas 1 patient in each group developed grade 4 renal toxicity. Regarding hypocalcaemia, 1 patient in the delayed group developed grade 3 hypocalcaemia and all other patients with hypocalcaemia were classified as grade 1–2.

### Switching strategy

In 40 patients, antiresorptive treatment was switched from bisphosphonates to denosumab due to progression of bone disease (*n* = 28, 70.0%) or after the first SRE (*n* = 12, 30.0%). This was the most common switching strategy since the change from denosumab to bisphosphonates was observed in only 2 patients whereas switching between different bisphosphonates was observed in 12 patients. Sixty patients continued with the same bisphosphonate therapy after the first SSE.

**Fig. 3:**
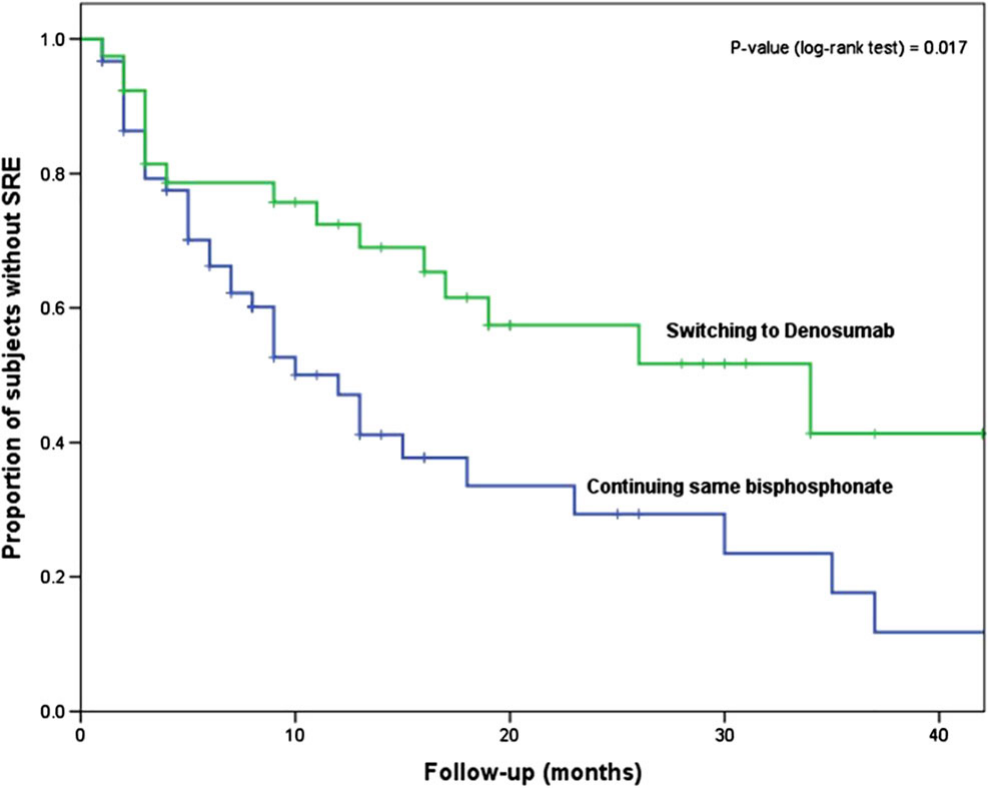
Kaplan-Meier curve for time from first SRE or skeletal disease progression to subsequent SRE in switching treatment group (from bisphosphonates to denosumab) vs. continuation of same bisphosphonate group

When we compared the time to SSE between the most common switching strategy (bisphosphonates to denosumab) and the continuation with the same bisphosphonate, we found a statistically significant longer time to SRE with switching strategy (Fig. 3). In the Cox proportional hazard model, switching strategy remained independently associated with longer time to SSE (HR 0.47, 95% CI 0.25–0.88, *p* value = 0.019) after adjustment for age, SSE at diagnosis, and number and type of bone metastases. However, no difference was observed in SMR (0.66 (SD 1.60) for switching strategy vs. 0.72 (SD 1.05) for continuing bisphosphonate; *p* value = 0.826).

### Treatment duration

The effect of treatment duration on outcome was studied in 121 patients who survived for more than 2 years. Of these patients, 51 (42%) continued antiresorptive treatment beyond 2 years whereas 70 (58%) discontinued.

Using the landmark approach at 24 months, we found that treatment continuation beyond 2 years was associated with longer time to first SSE after 2 years (*p* value = 0.013) as well as to subsequent SSE (*p* value = 0.039) compared with discontinuation of antiresorptive treatment. In the Cox regression proportional hazards model, antiresorptive treatment beyond 2 years was associated with longer time to SRE (HR 0.41; 95% CI 0.19–0.93) after adjustment for age, sex, Charlson comorbidity index, ECOG performance status, SSE at diagnosis, and number and type of bone metastases.

The SMR was not significantly different between the two treatment groups (0.14 (SD 0.63) SRE/year for treatment continuation beyond 2 years group vs. 0.27 (SD 0.41) SSE/year for treatment discontinuation group, *p* value = 0.179).

**Fig. 4:**
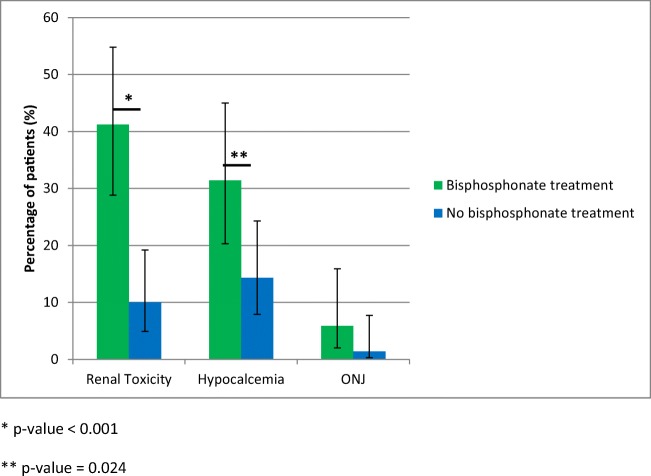
Toxicity rate for antiresorptive treatment-specific toxicities in patients with treatment continuation beyond 2 years vs. treatment discontinuation at 2 years

Regarding toxicity, patients in the treatment continuation beyond 2-year group had higher rate of renal toxicity (41.2% vs. 10%, *p* value < 0.001) and hypocalcaemia (31.4% vs. 14.2%, *p* value = 0.024) compared with treatment discontinuation group (Fig. 4). Two patients in each group developed an irreversible stage IV renal failure with Glomerular Filtration Rate (GFR) < 30 ml/min/1.73m^2^ whereas no patient required hospitalization due to severe hypocalcaemia. A numerical but not statistically significant difference in ONJ was observed between the two groups (*n* = 1 patient; 1.4% in the continuation group vs. *n* = 6 patients; 11.8% in the discontinuation group, *p* value = 0.176).

Only 35 patients continued antiresorptive treatment beyond 3 years; as a result, the number of patients was too small to perform any further analyses.

## Discussion

In our study cohort of 255 consecutive patients with metastatic cancer treated with antiresorptive treatment, we investigated the optimal use of antiresorptive agents through three clinical questions. We found that time to antiresorptive treatment initiation did not influence treatment outcome. In addition, a switching strategy from bisphosphonates to denosumab at skeletal disease progression or SRE reduced the risk for new SRE compared with continuing with bisphosphonate treatment. Furthermore, treatment continuation for more than 2 years had a positive impact on the risk for SSE. However, our results should be interpreted with caution considering the study limitations.

The first clinical question we tried to answer was whether time to antiresorptive treatment initiation upon diagnosis of bone metastases influences the treatment effect. The current treatment recommendation is to initiate antiresorptive treatment directly after the diagnosis of bone metastases [10–13]. This recommendation is, however, not supported by clinical evidence but rather reflects how the pivotal trials on antiresorptive treatment in metastatic setting were designed [9]. In clinical practice, delays in starting antiresorptive treatment are relatively common for instance in patients with poor dental hygiene that are in need for dental care before treatment initiation. Our study is the first that specifically address this clinical question. We found no detrimental effect on the time to SSE or the incidence of SSE in the delayed group (median time 7 months) compared with the direct group. Our results are hypothesis-generating and support further prospective studies investigating a more individualized approach on the time to antiresorptive treatment initiation. The second clinical question was whether switching strategy from bisphosphonates to denosumab in case of skeletal disease progression or SSE is more effective than continuation with bisphosphonates. We found a benefit for switching strategy in terms of time to SSE. However, due to the small sample size, our study was unable to investigate whether a switching strategy from denosumab to bisphosphonates could be beneficial as well in comparison with continuation of denosumab. Switching strategy has been investigated in some randomized phase II studies using surrogate endpoints as changes in bone turnover markers but without clinically relevant outcomes and found indirect evidence that the switching approach might be more effective [16–18]. Our results support the hypothesis that has been tested by these studies in a real-world setting by using clinically relevant outcomes such as time to SSE. Our results are also in accordance with indirect evidence from pivotal randomized studies about denosumab in which time to subsequent SRE in patients treated with denosumab (that could correspond to the switching strategy to denosumab after SRE in our study) was significantly longer than in patients treatment with bisphosphonates (corresponds to continuation of bisphosphonates after SRE in our study) [19]. Based on the current evidence, the switching strategy has a theoretical and early clinical potential to be the preferred treatment strategy in case of disease progression but further studies with clinically relevant outcomes are necessary.

The third clinical question was the treatment duration of antiresorptive treatment and whether continuing antiresorptive treatment for more than 2 years would add any clinically significant benefit. To minimize the risk for immortal time bias, we used the landmark approach and chose 2 years as landmark time, i.e., we included in the analysis patients that survived for at least 2 years and divided the patients into two groups based on the exposure to antiresorptive treatment at the landmark time or not. Continuing antiresorptive treatment for more than 2 years seemed to be beneficial in terms of time to first SSE after 2 years and time to subsequent SSE. As expected, the risk for drug-specific adverse events was higher in patients with treatment continuation of more than 2 years but we found no significant difference in serious adverse events (severe renal impairment, osteonecrosis of the jaw, hospitalization due to hypocalcaemia) or irreversible adverse events (need for renal replacement therapy due to irreversible renal impairment). Our results are in line with prior studies in which long-term use of antiresorptive treatment was found to be effective with increased, but manageable, toxicity [20–23]. Combining our results on the clinical benefit of continuing antiresorptive treatment for more than 2 years with recent evidence that enables a reduction in the dosing frequency of zoledronic acid to every 3 months [24–26], a reasonable treatment approach would be to continue with infusion every 3 months for more than 2 years.

An interesting observation from our study cohort was that a considerably large number of patients received antiresorptive treatment until their death. Current recommendations suggest that antiresorptive treatment should be continued until a substantial decline in the patient’s general performance status is detected and the reason behind this recommendation is the potential analgesic effect of such agents. Considering the lack of high-quality evidence on the analgesic role of antiresorptive treatment [27], the well-documented risk for aggressive non-beneficial cancer treatment at the end-of-life [28, 29], and the risk for adverse events, future studies should investigate the potential role of antiresorptive treatment at the end-of-life care.

There are several limitations in our study that need to be taken into account when interpreting the results. First, the retrospective nature of the study makes the results prone to selection and misclassification bias. Furthermore, the relative small sample size, especially in subgroups, did not enable further analyses that could shed light on other clinically relevant aspects on antiresorptive treatment, e.g., the potential benefit of such treatment for more than 3 years, the identification of subgroups in which antiresorptive treatment should be initiated directly, and the potential effect of switching from denosumab to bisphosphonates after disease progression or SSE. Finally, whether adverse events were related only to antiresorptive treatment or whether there were other factors that led to adverse events could not be retrospectively defined.

The current treatment approach with antiresorptive therapy in patients with bone metastases is based on the “one-size-fits-all” concept. Considering the obvious limitations of our study, our results are hypothesis-generating but support the need for further studies to better optimize the antiresorptive treatment in patients with bone metastases. Based on our results and current literature, antiresorptive treatment is an important supportive cancer therapy that should be offered in patients with bone metastases. Some hypotheses towards a more individualized approach on antiresorptive treatment are supported by our results including the lack of detrimental effect when the treatment is delayed, the potential benefit of switching strategy after skeletal disease progression or SSE, and the benefit of continuing antiresorptive treatment beyond 2 years. Future efforts should be made to a more personalized antiresorptive treatment approach regarding the choice of antiresorptive agent, the time to treatment initiation, the switching strategy, and the duration of treatment.
